# Genetic counselors' awareness and attitudes regarding gene therapies

**DOI:** 10.1002/jgc4.1953

**Published:** 2024-07-23

**Authors:** Chelsey Walsh, Andrea L. Durst, Damara Ortiz, Rachel G. Miller, Michelle Alabek

**Affiliations:** ^1^ Department of Human Genetics, Graduate School of Public Health Genetic Counseling Program, University of Pittsburgh Pittsburgh Pennsylvania USA; ^2^ Northwestern Medicine Winfield Illinois USA; ^3^ School of Medicine University of Pittsburgh Pittsburgh Pennsylvania USA; ^4^ UPMC Children's Hospital of Pittsburgh Pittsburgh Pennsylvania USA; ^5^ Department of Epidemiology, School of Public Health University of Pittsburgh Pittsburgh Pennsylvania USA; ^6^ UPMC Vision Institute Pittsburgh Pennsylvania USA

**Keywords:** education, gene therapy, genetic counseling, genetic counselors, treatment

## Abstract

There are 10 gene therapies (GTs) for hereditary conditions that are currently approved by the Food and Drug Administration (FDA). While prior research demonstrates that the majority of healthcare providers lack knowledge regarding GTs, this has not been explored within the genetic counseling profession. The authors hypothesize that the availability of GTs impacts the genetic counseling profession and that there is variable awareness on this topic among genetic counselors (GCs). We conducted a survey to assess GCs' familiarity with, comfort with, and frequency of discussing FDA‐approved GTs at the time of the survey, as well as GCs' perceived impact of and educational experiences related to GT. The survey was distributed through listservs and word of mouth from January through March 2021. One hundred of the 109 responses met eligibility criteria. Respondents were more familiar with onasemnogene abeparvovec‐xioi (ZOLGENSMA; Novartis Gene Therapies, Inc., Durham, NC, USA) than voretigene neparvovec‐rzyl (LUXTURNA; Spark Therapeutics, Inc., Philadelphia, PA, USA; *p* < 0.001). Familiarity with, comfort with, and frequency of discussing both GTs varied by specialty but not by years of experience. Fifty‐nine percent of respondents (58/98) reported that GTs impact their work, with differences by specialty but not by years of experience. The majority of respondents (93%; 90/97) felt that GCs should be comfortable discussing GTs with patients, and most respondents (83%; 79/95) were interested in additional GT training. Only 38% of respondents (36/95) recalled GT being included in their genetic counseling training program's curriculum, which may be skewed by recent growth of this field. Our results suggest that GCs feel that GTs impact their practice, have discrepant awareness and comfort in this area, and desire additional training on this topic. Further investigation into the actual impact and models for addressing training is warranted and will be critical as the number of approved GTs increases.

**What is known about this topic**

Healthcare providers report a lack of knowledge regarding gene therapy, and they express a desire for additional educational opportunities.

**What this paper adds to the topic**

To our knowledge, this is the first study assessing training, awareness, and experiences regarding gene therapies among genetic counselors. This paper begins to characterize the impact of novel therapies on the genetic counseling profession and highlights the need for additional research, training, and education on this topic.

## 1 INTRODUCTION

Historically, the medical care of genetic disease has focused on symptom management, including dietary modifications and medications. Scientific discoveries and improved genetic technologies have led to the development of disease‐specific treatments for these conditions, such as enzyme replacement therapies and gene therapies, increasing the options available for treating genetic diseases. Gene therapy is “the use of genetic material to treat or prevent a disease” (American Society of Gene and Cell Therapy, 2022). This term encompasses a diverse group of treatments that can be used to target hereditary and acquired diseases with an array of differences, including type of material delivered, vector used, method of administration, and mechanism of action. In December 2017, the FDA approved the first gene therapy for a hereditary condition: voretigene neparvovec‐rzyl (LUXTURNA; Spark Therapeutics, Inc., Philadelphia, PA, USA; FDA, 2019). This adeno‐associated virus (AAV) gene therapy is delivered via subretinal injection to treat patients with retinal dystrophy due to biallelic pathogenic variants in *RPE65*. In May 2019, the FDA approved the second gene therapy for a hereditary condition, onasemnogene abeparvovec‐xioi (ZOLGENSMA; Novartis Gene Therapies, Inc., Durham, NC, USA; FDA, 2019; Mendell et al., 2017). This AAV gene therapy is delivered via intravenous infusion to treat patients with spinal muscular atrophy (SMA). While further follow‐up is being done to determine a complete profile of long‐term effects of these treatments, voretigene neparvovec‐rzyl^#^ and onasemnogene abeparvovec‐xioi^^^ have been shown to positively impact the progressive natural history of their respective disease.

As of January 2024, the FDA has approved eight additional gene therapies for hereditary conditions, including beta‐thalassemia, cerebral adrenoleukodystrophy, Duchenne muscular dystrophy, dystrophic epidermolysis bullosa, hemophilia A, hemophilia B, and sickle cell anemia. Additionally, numerous gene therapies are in pre‐clinical research and clinical trials, and it has been estimated that more than 50,000 non‐oncology patients will be treated with gene therapy by 2030 (Barrett et al., 2023; Young et al., 2022). These novel therapies represent a paradigm shift in the treatment and management of genetic diseases.

Limited data are available regarding the readiness of healthcare providers and medical systems to integrate these therapies into clinical care. One recent study found that nearly two‐thirds of healthcare providers were unaware of current FDA‐approved gene therapies (Rare Neurological Disease Special Report, 2020). The majority of these respondents identified “limited knowledge among healthcare professionals” as a barrier to gene therapy for patients, and approximately one‐third of respondents reported that they had not sought out any type of education on gene therapies or clinical trials (Rare Neurological Disease Special Report, 2020). None of the respondents in this survey were genetic counselors (GCs) or medical geneticists. Another recent survey of healthcare providers regarding gene therapy research for inherited metabolic disorders (IMDs) found that, while nearly all respondents were aware of gene therapy, they had generally “low confidence” in being able to discuss, educate, or counsel patients and their families about the topic, and the majority were interested in additional education (Hansen et al., 2022). These studies provide evidence that increasing healthcare provider knowledge and comfort will be essential as more gene therapies become available.

A similar discrepancy between familiarity and comfort level discussing treatments has been previously documented in studies of prenatal GCs regarding new treatments for SMA and cystic fibrosis (CF) (Elsas et al., 2017; Zettler et al., 2022). Both studies suggest that GCs need additional resources to be prepared to apply their skills in the context of these therapies (Elsas et al., 2017; Zettler et al., 2022). While these studies document a gap in knowledge regarding novel treatments for hereditary conditions among prenatal GCs, they failed to assess the profession as a whole and did not explore gene therapies.

A recent publication resulting from an advisory board of medical geneticists addressed the need for both their profession and healthcare systems to prepare for the increasing number of gene therapies, highlighting a role for GCs (Vockley et al., 2023). Previous studies have determined that GCs are frequently assessing their ever‐changing professional relationships to other providers (Bensend et al., 2014) and their roles on a healthcare team (Veach et al., 2001). GCs describing ideal ways to provide “genetic services” suggested that a multidisciplinary approach would be most beneficial for patient care and could avoid negative outcomes (Bensend et al., 2014). Based on the training of GCs, this profession is well‐suited to contribute a unique skillset to the multidisciplinary care of patients potentially eligible for a gene therapy. To maximize this contribution, the genetic counseling profession will need to understand whether the availability of gene therapies is impacting genetic counselor practice and to prepare our profession to be a key stakeholder in the process of patients accessing gene therapies. This has not been previously explored to our knowledge. There are currently no competencies, practice guidelines, or educational tools specific to GCs regarding gene therapy. The accreditation guidelines for GC graduate programs outline topics to be covered during training (Accreditation Council for Genetic Counseling (ACGC), 2023b). These guidelines do not specifically mention gene therapy. However, they do include topics such as case management in the context of different genetic counseling specialties (B.2.1.3.c), which could be applied to awareness of gene therapy (ACGC, 2023b). Several Accreditation Council for Genetic Counseling (ACGC) Practice Based Competencies (ACGC, 2023a) highlight specific skills that could be applicable to support patients considering gene therapies, including addressing psychosocial concerns that impact a patient's understanding of their condition and subsequent decision‐making (sub‐competencies 3.a, 3.c, and 3.d.) and communicating information to patients (competency 4.). While these may be more relevant to GCs practicing in subspecialties where gene therapies are available, there are also several competencies that suggest that baseline knowledge and awareness of gene therapy is important for all GCs, regardless of area of practice. These include understanding genetic conditions and testing methods (competency 1., sub‐competency 1.c.), as well as engaging in current research (sub‐competency 5.c.). Competencies 6. and 7. address the scope of GC practice and how GCs fit into a healthcare team, which are continuously evolving. Despite this, it is unclear whether a standardized baseline knowledge of gene therapy exists among GCs. GC‐specific educational resources regarding gene therapy do not currently exist but could promote standard awareness of and comfort with gene therapy, positively impacting both new graduates and established practicing GCs.

In this study, we perform an initial exploratory evaluation of the intersect between gene therapy and the genetic counseling profession. The primary aims were to document and compare GC familiarity with, comfort level, and frequency of discussing FDA‐approved gene therapies by specialty area and by years of experience. Secondary aims included exploring the perceived impact of gene therapies on GC practice and GC educational experiences and desires related to gene therapy.

## 2 METHODS, SURVEY AND STUDY DESIGN, AND STATISTICAL ANALYSIS

A survey including 22 multiple choice, open‐ended, and Likert‐scale questions was developed by the authors of this study with varied expertise in genetic counseling, medical genetics, gene therapy, and academics. The survey was piloted by the authors and three GCs not affiliated with the study. Questions were designed to assess self‐reported perceptions of the respondents. Two FDA‐approved gene therapies for hereditary conditions existed at the time of survey development and distribution. Only proprietary drug names (i.e. LUXTURNA, ZOLGENSMA) were used in the survey for easier readability. Recruitment for this observational, cross‐sectional study occurred through non‐probability sampling methods. An anonymous Qualtrics survey link in English was distributed to the National Society of Genetic Counselors (NSGC) student research listserv and was posted on the NSGC website. In an effort to ensure representation of GCs working in subspecialties with active gene therapy landscapes, the same survey link was distributed to an ophthalmology GC list serve, a hematology GC listserv, a metabolic providers listserv, and directly to 3 GCs known to the authors to work in relevant industry positions. Responses were collected from January 2021 to March 2021. Individuals who were a board‐certified or board‐eligible GC working in North America at the time of the survey were eligible, which is estimated to be approximately 6000 individuals (Abacan et al., 2019; Lambert et al., 2022; NSGC, 2021a). Four individuals were determined to be ineligible after indicating that they were not an ABGC or CAGC board‐certified or board‐eligible genetic counselor and thus were not able to complete the survey. The following 3 survey questions addressing the primary aims of the study were required to be completed for a response to be included in the analytic sample: “Q6: How familiar are you with the following gene therapies?”, “Q7: How comfortable are you discussing the following gene therapies with a patient?”, and “Q8: How often do you discuss the following gene therapies with a patient?”. Five respondents did not answer questions 6, 7, or 8 and were excluded from analysis. The other questions were assessing secondary endpoints and were thus considered optional; however, the completion rate was high, with all items having less than 5 respondents with missing data. Therefore, all analyses were performed using a complete‐case approach. This survey was determined to be exempt by the University of Pittsburgh Institutional Review Board (IRB).

Descriptive statistics were calculated as *n* (%) by years of experience and separately by specialty area. The within‐respondent familiarity with voretigene neparvovec‐rzyl^#^ compared to onasemnogene abeparvovec‐xioi^^^ was tested using Bowker's test of symmetry. The distributions of Likert‐scale responses were compared across years of experience categories using Spearman's rank correlation. For analyses by specialty area, the distribution of responses for each specific specialty area was compared to all other specialties combined, in a pairwise manner, using the Cochran's test for trend. Bowker's test of symmetry was also used to evaluate the association between comfort level and frequency of discussing each gene therapy. A *p*‐value of <0.05 was considered statistically significant for all analyses. Analyses were performed using SAS 9.4 (SAS Institute Inc., Cary, NC) and Qualtrics.

## 3 RESULTS

### 3.1 Demographics

One hundred respondents met inclusion criteria. Based on the estimate of approximately 6000 eligible board‐certified or board‐eligible GCs in North America, respondents represent approximately 1.67% of eligible GCs (Abacan et al., 2019; Lambert et al., 2022; NSGC, 2021a, 2021b). Distributions of years of experience and areas of specialty are summarized in Table 1. Years of experience was skewed toward more recent graduates, with the majority reporting they graduated five or less years ago (56%; 55/98). Respondents were able to select multiple areas of current and prior specialties, with pediatrics being the most common. There was representation from specialties with active gene therapy landscapes, including IMDs, neurology, ophthalmology, and hematology.

**TABLE 1:** Self‐reported demographics of respondents.

	Response %
Years of experience (*n* = 98)	
<1 year	13%
1–5 years	43%
6–10 years	14%
11–15 years	9%
16–20 years	10%
21–25 years	6%
25+ years	4%
Specialty (*n* = 98)	
Pediatrics	51%
Metabolic Disorders	36%
Research	30%
Cancer	28%
Prenatal	28%
Neurology	26%
Ophthalmology	21%
Laboratory	14%
Cardiology	14%
Hematology	11%
Other	15%

*Note*: This table reflects the answers of the 98 respondents who answered questions regarding their demographics; 2 respondents did not answer these questions. Respondents were able to select multiple specialties and were asked to include any specialties from their entire career; therefore, percentages will not add up to 100. Respondents were able to give short answers to the “other” option. Short answers included translational medicine, public health, PGD, newborn screening, neurofibromatosis, mitochondrial disease, inpatient, skeletal, report writing, adult general, PAG, health IT, consulting, and industry.

### 3.2 Familiarity, comfort level, and frequency of discussing

Overall, respondents were less familiar with voretigene neparvovec‐rzyl^#^ than onasemnogene abeparvovec‐xioi^^^ (*p* < 0.001; see Table 2). There was no correlation between years of experience and familiarity with voretigene neparvovec‐rzyl^#^ (*r* = −0.11, *p* = 0.29) or onasemnogene abeparvovec‐xioi^^^ (*r* = −0.03, *p* = 0.76). Familiarity with voretigene neparvovec‐rzyl^#^ was higher among respondents with experience in ophthalmology (*p* < 0.001) and research (*p* = 0.01) and was lower among respondents with experience in cancer (*p* = 0.01), prenatal (*p* = 0.01), and metabolic conditions (*p* = 0.01). Familiarity with onasemnogene abeparvovec‐xioi^^^ was higher among respondents with experience in neurology (*p* < 0.001) and lower among respondents with experience in cancer (*p* = 0.002). Respondents most commonly described themselves as “slightly comfortable” discussing gene therapies with patients, with 45% and 51% of respondents selecting this option for voretigene neparvovec‐rzyl^#^ and onasemnogene abeparvovec‐xioi^^^, respectively (see Table 2). There was no correlation between years of experience and comfort discussing voretigene neparvovec‐rzyl^#^ (*r* = −0.12, *p* = 0.24) or onasemnogene abeparvovec‐xioi^^^ (*r* = −0.07, *p* = 0.49). Comfort discussing voretigene neparvovec‐rzyl^#^ was higher among respondents with experience in ophthalmology (*p* < 0.001) and research (*p* < 0.001) and lower among respondents with experience in prenatal (*p* = 0.04) and metabolic specialties (*p* < 0.001). Comfort discussing onasemnogene abeparvovec‐xioi^^^ was higher among those with experience in neurology (*p* < 0.001).

**TABLE 2:** GC familiarity with, comfort discussing, and frequency of discussing gene therapy.

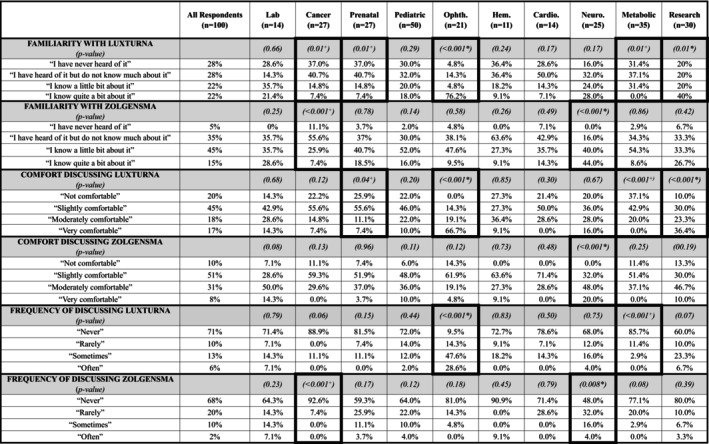

*Note*: The responses to Likert‐scale questions 6–8 are summarized in this table. The first column displays the distribution of all 100 respondents. Subsequent columns display the distributions by self‐reported specialty. Respondents could select more than 1 specialty, so the total answers for specialty do not equal 100. Specialties with statistically significant findings are depicted by bold rectangles. Since we referenced the therapies as Luxturna and Zolgensma in the survey, we referenced them as such in this table. Luxturna = voretigene neparvovec‐rzyl and Zolgensma = abeparvovec‐xioi.

Abbreviations: Cardio., cardiology; Hem., hematology; Neuro., neurology; Ophth., ophthalmology.

* *p*‐Value indicating a specialty that is more familiar with, more comfortable discussing, or more frequently discussing the gene therapy compared to all other specialties.

^+^ *p*‐Value indicating a specialty that is less familiar with, less comfortable discussing, or less frequently discussing the gene therapy compared to all other specialties.

The majority of respondents indicated that they “never” or “rarely” discussed voretigene neparvovec‐rzyl^#^ (81%; 81/100) or onasemnogene abeparvovec‐xioi^^^ (88%; 88/100) with patients (see Table 2). Only 6% (6/100) and 2% (2/100) of respondents indicated that they “often” discussed voretigene neparvovec‐rzyl^#^ and onasemnogene abeparvovec‐xioi^^^, respectively. There was no correlation between years of experience and frequency of discussing voretigene neparvovec‐rzyl^#^ (*r* = −0.009, *p* = 0.93) or onasemnogene abeparvovec‐xioi^^^ (*r* = 0.03, *p* = 0.76). Frequency of discussing voretigene neparvovec‐rzyl^#^ was higher among those with experience in ophthalmology (*p* < 0.001) and lower among those with experience in metabolic disorders (*p* = 0.006). Frequency of discussing onasemnogene abeparvovec‐xioi^^^ was higher among respondents with experience in neurology (*p* = 0.008) and lower among those with experience in cancer (*p* = 0.003). There was a significant association between the level of comfort with and the frequency of discussing each therapy for both voretigene neparvovec‐rzyl^#^ (*p* < 0.001) and onasemnogene abeparvovec‐xioi^^^ (*p* < 0.001). The sample size was too small to further analyze this association by specialty or years of experience.

Respondents with experience in research were more familiar with and more comfortable discussing voretigene neparvovec‐rzyl^#^ than those without experience in research. Given that this difference was not identified with respect to onasemnogene abeparvovec‐xioi^^^, we investigated the possibility of a false correlation due to respondents with experience in both research and ophthalmology. While not statistically significant, there was a trend that those reporting experience in research were more likely to report experience in ophthalmology compared to those who did not report research experience (*p* = 0.06). However, there was also a trend that those reporting research experience and no ophthalmology experience were still more likely to report greater familiarity with voretigene neparvovec‐rzyl^#^ than those not reporting research experience (*p* = 0.07). Given that these did not reach statistical significance, a correlation between familiarity with and comfort discussing voretigene neparvovec‐rzyl^#^ among those with research experience should be interpreted with caution.

### 3.3 Importance and perceived impact

Seventy‐seven of 97 respondents (79%) felt that it is “extremely important” or “very important” for GCs to know about gene therapies, with an additional 17 respondents (18%) reporting that it was “moderately important.” While not statistically significant, importance tended to be rated higher as years of experience increased (*r* = 0.15, *p* = 0.09). Importance was rated higher among respondents with experience in research (*p* = 0.02). Of the 97 respondents who answered which type(s) of providers should feel comfortable discussing gene therapies with patients, “geneticist” (98%; *n* = 95), “genetic counselor” (93%; *n* = 90), and a “physician managing patients who potentially could be treated with gene therapy (ex: neurologist or ophthalmologist)” (91%; *n* = 88) were the most common answers. These percentages total greater than 100%, as respondents were allowed to choose multiple provider types. All respondents who chose “genetic counselor” also selected at least one other provider type.

**FIGURE 1:**
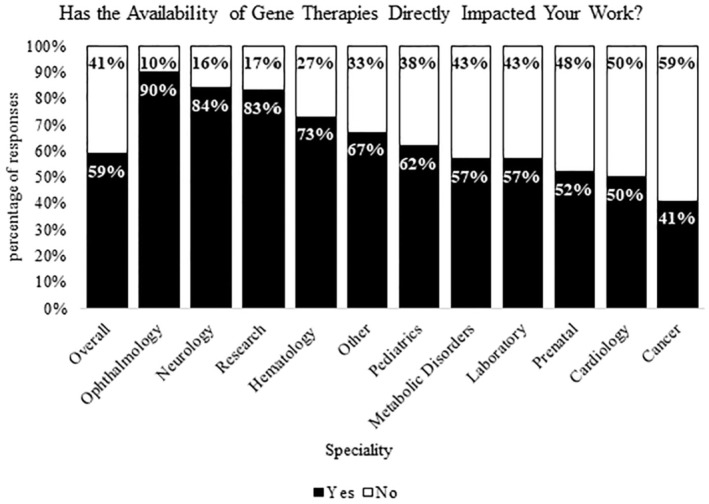
Self‐reported perceived impact of gene therapy on GC work/practice. Respondents were asked if the availability of gene therapies has directly impacted their work as a GC. Based on the survey design, this is a self‐reported perception by respondents. The first bar depicts responses from the entire cohort (*n* = 100). Subsequent bars depict responses within each specialty, ordered from highest agreeing to lowest agreeing. Black = yes. White = no. Respondents could select more than 1 specialty, so the total answers for specialty do not equal 100. Respondent specialties were Ophthalmology (*n* = 21), Neurology (*n* = 25), Research (*n* = 30), Hematology (*n* = 11), Other (*n* = 15), Pediatrics (*n* = 50), Metabolic Disorders (*n* = 35), Laboratory (*n* = 14), Prenatal (*n* = 27), Cardiology (*n* = 14), and Cancer (*n* = 27).

The majority of respondents indicated that they perceive the availability of gene therapy has directly impacted their counseling practice (see Figure 1). Perceived impact did not differ by years of experience (*p* = 0.21). Respondents with experience in cancer were less likely to report a perceived impact (*p* = 0.02) than other specialties. Perceived impact was more likely to be reported among respondents with experience in ophthalmology (*p* = 0.001), neurology (*p* = 0.003), and research (*p* = 0.001).

### 3.4 Education and training

**FIGURE 2:**
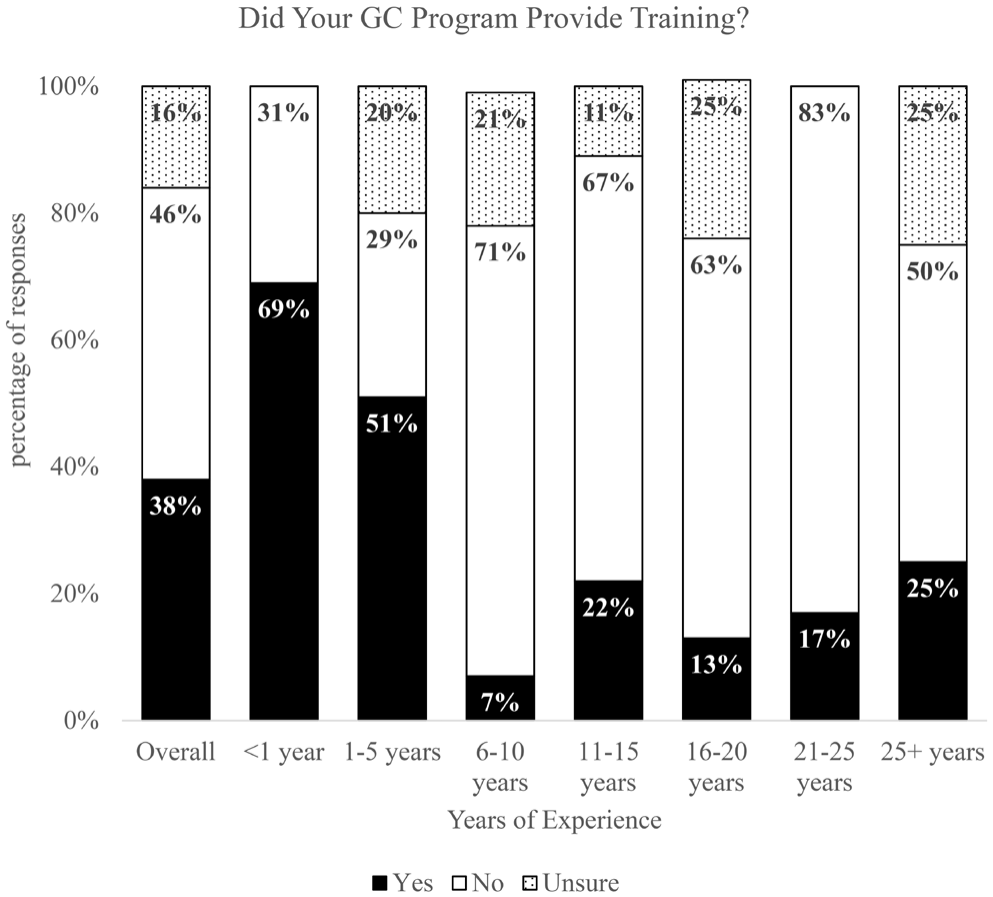
Training provided by genetic counseling programs broken down by years of experience. Respondents were asked if training regarding gene therapy was included in their genetic counseling graduate program. Based on the survey design, this is a self‐reported recollection by respondents. The first bar depicts responses from the entire cohort (*n* = 100). Subsequent bars depict responses by years of experience. Black = yes. White = no. Spotted = unsure. Respondent years of experience was less than 1 year (*n* = 13), 1–5 years (*n* = 42), 6–10 years (*n* = 14), 11–15 years (*n* = 9), 16–20 years (*n* = 10), 21–25 years (*n* = 6), more than 25 years (*n* = 4), and did not answer (*n* = 2).

Of the 95 respondents who answered the questions assessing prior education and training regarding gene therapy, only 38% of respondents (*n* = 36) recalled that their genetic counseling program provided training covering gene therapies (see Figure 2). Recalled inclusion of gene therapy training differed by years of experience (*p* = 0.001); respondents with less than 1 year and 1–5 years of experience reported inclusion more frequently (see Figure 2). Inclusion of gene therapy training did not differ by specialty (all *p*‐values >0.05).

Fifty‐nine of the 95 respondents (62%) reported that they attended training or continuing education regarding gene therapies. The majority of respondents (83%; 79/95) indicated that they were interested in additional training. Interest did not differ by years of experience (*p* = 0.16). There was no statistically significant difference in interest by specialty (all *p*‐values >0.05). Preferences regarding hosts and formats for future educational opportunities are outlined in Table 3.

**TABLE 3:** GC preferences for future gene therapy education.

	Response %
Source or provider of training^a^ (*n* = 78)	
NSGC	78%
Scientific journal articles	77%
Other professional organizational training	71%
Drug manufacturers	58%
Training through your workplace	45%
FDA	36%
A patient advocacy group	39%
Word of mouth/discussions with colleagues	19%
Other	3%
Popular media (TV, newspaper, magazines, etc.)	0%
Method of training^b^ (*n* = 79)	
Seminar/lecture	96%
Online training	67%
Brochure/written literature	44%
Workplace discussion	37%
Sales pitch	1%
Other	1%

*Note*: Respondent preferences for future trainings are outlined in this table. Percentages total >100%, as respondents were able to select multiple options.

^a^ A total of 324 preferences regarding source of provider of training were received from 78 respondents.

^b^ A total of 95 preferences regarding method of training were received from 79 respondents.

## 4 DISCUSSION

To our knowledge, this is the first study investigating the intersect of FDA‐approved gene therapies and the genetic counseling profession. Our data suggest that GCs are already feeling that the availability of these therapies is impacting GC practice. Additionally, our study found that GCs feel that the profession should be aware of gene therapies and that involvement with gene therapies is within GC scope of practice. These perceptions were identified with only 2 FDA‐approved gene replacement therapies being available at the time of surveying. Given the rapid advancement of this field of therapeutics, it is logical to presume that this perception among GCs will continue to grow. It is important to recognize that our study did not assess GC technical knowledge of gene therapies or advancements in the field. These topics, as well as trends in perceptions overtime, will be important to evaluate moving forward.

While it may seem obvious that GCs practicing in specialty areas with available gene therapies perceive that gene therapies are impacting their practice, our study suggests a perceived impact across all specialties. While actual impact of gene therapies on the GC profession was outside of the scope of this study, one can hypothesize possible ways these therapies are impacting the broader profession, such as more awareness among the general public resulting in questions from patients with genetic diseases, GCs addressing natural history and available treatment options with patients after carrier testing or newborn screening, or changes in workflow. Prior studies have reported that GCs feel it is appropriate to discuss available treatments for genetic conditions as early as the prenatal setting and that GCs may be the first provider to have a general discussion with parents about the disease, treatment, and management options (Elsas et al., 2017). While these roles may also apply to the treatment category of gene therapies, further studies are warranted to investigate and qualify the actual impact presented by this group of novel treatments on the genetic counseling profession. Given the dynamic nature of these therapies, it will be important for both the perceived and actual impacts to be continuously evaluated over time.

In our study, approximately two‐thirds of respondents in 2021 had heard of at least one of the two FDA‐approved therapies available at the time of survey. This falls in the range of other prior studies of prenatal GCs, where awareness of novel therapies for genetic conditions ranged from 20% to 95% (Elsas et al., 2017; Zettler et al., 2022). Despite voretigene neparvovec‐rzyl^#^ being the first FDA‐approved gene therapy, GCs were more aware of onasemnogene abeparvovec‐xioi^^^. This could be related to prevalence of the conditions treated by these therapies, with SMA estimated to be 3 to 4 times more prevalent than *RPE65*‐related inherited retinal dystrophy in the United States (U.S. and World Population Clock, 2023; Lloyd et al., 2019; Verhaart et al., 2017). It could also be due in part to other factors, including fewer GCs working in ophthalmology than neurology, the availability of multiple SMA treatments, or the recommendation to include SMA on newborn screens in 2018 (HRSA, 2023; NSGC, 2021a; NSGC, 2021b). Additionally, SMA is a standard offering on all carrier screening panels, while *RPE65* is only on expanded panels that might not be frequently ordered (ACOG's Committee on Genetics, 2017). The authors found it interesting that there was low awareness of the first FDA‐approved gene therapy for a hereditary condition within a profession focused on patients with genetic diseases.

Consistent with what has been previously reported among non‐genetics providers, our data suggest that GCs working in specialty areas with active gene therapy landscapes are more likely to be aware of and comfortable discussing gene therapy with patients (Rare Neurological Disease Special Report, 2020). Specifically, GCs with experience in ophthalmology and neurology had higher familiarity with, comfort discussing, and frequency of discussing the therapy relevant to their specialty and were also more likely to report that the availability of gene therapy was impacting their practice. Interestingly, this was not the case among other specialties with active gene therapy landscapes at the time of the survey (i.e. hematology, IMDs). Possible explanations include that GCs in these specialties have lower awareness of relevant clinical trials or that patients in these specialties are not directing questions about gene therapies to GCs; however, this warrants further investigation, as our data are unable to discern whether a respondent was actively working in one of these areas at the time of the survey. Several additional differences were noted between specialties, most notably cancer and research, again warranting further investigation.

Respondents with experience in cancer were less familiar with both approved gene therapies and were less likely to report that the availability of gene therapy impacted their work. While this may be expected in GCs without experience in ophthalmology or neurology, these were not statistically significant for any other specialty. There are multiple approved gene therapies in oncology for non‐hereditary diagnoses (FDA, 2019). Therefore, it was unexpected to the authors that this was the only specialty less likely to report that they perceived gene therapy impacted their work. This may be due to these therapies not specifically targeting hereditary cancer syndromes; however, further exploration into the role and impact of cancer GCs related to gene therapy may be beneficial.

Respondents with experience in research were more likely to feel that the availability of gene therapies impacted their work. This was the only specialty to report higher importance for GCs to be knowledgeable about gene therapies. This could be due to the volume of gene therapies in pre‐clinical and clinical trials and the role research GCs are playing to support these efforts; however, this warrants further exploration.

While the sub‐optimal familiarity with and comfort discussing gene therapy may only seem relevant in certain specialties at this time, it is important to address this knowledge gap on a broader scale in preparation for more therapies becoming approved. Our study found that the majority of respondents feel that GCs should be knowledgeable about gene therapies and desire additional gene therapy education, regardless of years of experience or specialty. Nearly all respondents in our survey felt that GCs should be comfortable discussing gene therapies with patients, but awareness of both treatments was higher than their respective comfort level. This supports Zettler's claim that, even with high awareness of an available treatment, further training may be needed to better prepare GCs to discuss this treatment with patients (Zettler et al., 2022). Even GCs with experience in cancer, the only specialty that was less likely to report that gene therapies were impacting their work, were equally interested in education on this topic. This could represent anticipated impact of pipeline therapies throughout the genetic counseling profession or a general interest in any continuing education among GCs (i.e. natural history of disease, carrier testing, newborn screening, and targeted oncology treatments). Regardless, this demonstrates an opportunity for improved knowledge about gene therapies among GCs across all specialties. The rapid growth of this field highlights an urgency to develop an educational strategy for GCs to be knowledgeable of advances in this field that will likely impact their practice. Given the diversity of GC specialties and roles, a multi‐faceted approach including general and disease‐specific gene therapy topics and resources could be useful to address this knowledge gap; however, making specific recommendations for education is outside of the scope of this study and warrants further investigation.

Although gene therapy is not specifically addressed in ACGC standards, our data suggest that approximately one‐third of GCs responding to our survey recall receiving training on gene therapies through their graduate training program, with this being even more commonly reported among more recent graduates (ACGC, 2023b). The cause of this trend is unknown, but the authors speculate that it could suggest that some programs may have added gene therapy to their curriculum over the last 5 years. This could be a result of the FDA approvals or it could represent recall bias. Additional studies of GC program content could be useful to investigate this topic further. While it is positive that more recent graduates report receiving training about gene therapy as part of their program, it also highlights a potential discrepancy in baseline knowledge among GCs entering the profession. Our study only assessed recollection of training either occurring or not occurring, leaving us unable to comment on the content or consistency across programs. Incorporating gene therapy education into GC program curriculum presents an opportunity to establish baseline competency on the topic within the profession; however, given the extent of current curriculum requirements, the method for and feasibility of this idea must be explored.

Additional approaches will also need to be explored to improve the knowledge of currently practicing GCs. Nearly two‐thirds of our respondents reported that they had attended training or continuing education regarding gene therapies. This is far higher than the less than one‐third of general health providers who report attending such trainings (Rare Neurological Disease Special Report, 2020). Our survey did not define “previous training,” so it is difficult to determine whether this higher prevalence is an over‐ or under‐representation as a result of survey design. Despite a large proportion of respondents who reported attending prior trainings on gene therapy, GCs from every specialty answered that they would appreciate more education. Preferred opportunities for additional training are summarized in Table 3 and were similar to those suggested by other studies among GCs (Elsas et al., 2017; Zettler et al., 2022). Our data found that, even though GCs with experience in a specialty with an approved gene therapy have higher familiarity with and comfort discussing the relevant therapy, they still desire additional gene therapy education. This suggests that both general and specialty‐specific educational opportunities would be well‐received among practicing GCs. It is important to recognize that our study did not objectively measure knowledge of gene therapy among GCs. Studying this could further inform educational initiatives within the profession.

While our study found that GCs feel they should be comfortable discussing gene therapies with patients, this in no way suggests that GCs should be addressing this topic in isolation. Respondents felt that specialty physicians and medical geneticists should be involved in these discussions. This supports prior studies which found that gene therapy should be addressed by GCs in collaboration with other disciplines (Elsas et al., 2017; Zettler et al., 2022). Other healthcare providers (i.e. physicians, dieticians) also advocate that multidisciplinary practice will be essential with regard to gene therapy and that educational opportunities should be available to all types of providers working with these patients (Hansen et al., 2022). Further studies evaluating other stakeholders' (i.e. patients, physicians, pharmaceutical companies) perceptions of the role of GCs in this evolving field may be beneficial and could inform additional guidelines or training for GCs.

## 5 STUDY LIMITATIONS

Limitations to this study include recall bias, recruitment bias, and small sample size. As this study was intended to be exploratory, there were inherent statistical analysis limitations, including lack of predetermined sample size and no corrections for multiple testing. While the distribution of years of experience is similar to that of the 2021 Professional Status Survey, statistical comparison could not be performed due to discrepant response categories (NSGC, 2021a, 2021b). Minimal demographic variables were collected from respondents, which limits ability to assess representativeness of respondents compared to the overall profession. Other confounding variables were not able to be assessed, such as GCs having experience in multiple specialties or the level of exposure to gene therapy that GCs have on the job; these factors should be considered in development of future studies on this topic. Survey design did not differentiate between current and past specialty experience and did not assess GC training program attended, which limit our ability for further analysis related to these variables. Lastly, given the rapidly advancing nature of this field, these data collected in 2021 may not be fully representative of current professional trends.

## 6 CONCLUSION

This study provides baseline information about the experiences of GCs regarding the two initially FDA‐approved gene therapies for hereditary conditions. GCs feel that the availability of these gene therapies impacts their work. Although most respondents do not frequently discuss gene therapies with patients, the majority feel it is important for GCs to be knowledgeable about available gene therapies and to feel comfortable discussing gene therapies. While respondents want to be involved in multidisciplinary teams treating patients considering gene therapy, they desire additional training to be prepared to do so. Increased gene therapy education for GCs warrants a multi‐faceted approach to address current and future professional growth and demand.

## AUTHOR CONTRIBUTIONS

Chelsey Walsh, Andrea Durst, Damara Ortiz, and Michelle Alabek all significantly contributed to the conception and design of the work, analysis and interpretation of data, drafting and revising the paper, and final approval of the version to be published. Authors Walsh, Durst, Miller, and Alabek confirm that they had full access to all the data in the study and take responsibility for the integrity of the data and the accuracy of the data analysis. All of the authors gave final approval of this version to be published and agreed to be accountable for all aspects of the work in ensuring that questions related to the accuracy or integrity of any part of the work are appropriately investigated and resolved.

## CONFLICT OF INTEREST STATEMENT

Authors Walsh, Durst, Ortiz, Miller, and Alabek declare that they have no conflict of interest.

## ETHICS STATEMENTS

Human Studies and Informed Consent: Approval to conduct this human subject research was obtained by the University of Pittsburgh Institutional Review Board (STUDY20100152). All procedures followed were in accordance with the ethical standards of the responsible committee on human experimentation (institutional and national) and with the Helsinki Declaration of 1975, as revised in 2000. Informed consent was obtained from all patients for being included in the study.

Animal studies: No non‐human animal studies were carried out by the authors for this article.

## Supporting information


Appendix S1

## Data Availability

The data that support the findings of this study are available from the corresponding author upon reasonable request.
