# Supplementary material for: Genetic counselors' awareness and attitudes regarding gene therapies
Source: J Genet Couns. 2024 Jul 23;34(2):e1953. doi: 10.1002/jgc4.1953 (PMC11754531; doi:10.1002/jgc4.1953)
Supplement: Supplementary file 1 — Appendix S1 [file JGC4-34-0-s001.docx]

Gene Therapies Survey

Start of Block: Intro Statement

Q1 Thank you for your interest in this research study. This survey is meant to assess the current knowledge and attitudes that genetic counselors have regarding gene therapies.
Questions are centered on the two currently FDA approved gene therapies Zolgensma (onasemnogene abeparvovec-xioi) and Luxturna (voretigene neparvovec-rzyl). There will be opportunities to describe your experience with other gene therapies (such as those still in clinical trials) as well. 
The questionnaire should take between 10-20 minutes to complete. There are no foreseeable risks associated with this project, nor are there any direct benefits to you. This is a confidential questionnaire. Your participation is voluntary and you do not have to complete or submit the survey. 
This study is being conducted by Chelsey Walsh as part of a thesis requirement for the University of Pittsburgh Genetic Counseling Program. Chelsey can be reached at cnw30@pitt.edu if you have any questions.

End of Block: Intro Statement

Start of Block: Demographics

Q2 Are you an ABGC or CAGC board-certified or board-eligible genetic counselor?

- Yes (1)
- No (2)

Skip To: End of Survey If Q2 = No

Q3 How many years of experience do you have as a practicing genetic counselor?

- (1)
- 1-5 years (2)
- 6-10 years (3)
- 11-15 years (4)
- 16-20 years (5)
- 21-25 years (6)
- 25+ years (7)

Q4 Please check all scopes of practice you have worked in throughout your entire career:

- Laboratory (1)
- Cancer (2)
- Prenatal (3)
- Pediatrics (4)
- Ophthalmology (6)
- Hematology (7)
- Neuromuscular Disorders (8)
- Cardiology (9)
- Neurology (10)
- Metabolic Disorders (11)
- Research (12)
- Specialty Not Listed Above (14) ________________________________________________
- Other (industry, patient education, etc.) (13) ________________________________________________

End of Block: Demographics

Start of Block: Statement

Q5 **If you are genetic counselor that does not regularly see patients (i.e. laboratory or research), please substitute “patient” with “client” or “other healthcare provider” to answer the rest of the survey's questions.**

End of Block: Statement

Start of Block: Experiences

| 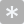 |
| --- |

Q6 How familiar are you with the following gene therapies?

|  |  | | | |
| --- | --- | --- | --- | --- |
|  | I have never heard of it. (1) | I have heard of it but I do not know much about it. (2) | I know a little bit about it. (3) | I know quite a bit about it. (4) |
| Zolgensma to treat Spinal Muscular Atrophy (2) |  |  |  |  |
| Luxturna to treat RPE65 Associated Inherited Retinal Dystrophy (3) |  |  |  |  |
| Other (please describe) (not required) (4) |  |  |  |  |

| 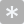 |
| --- |

Q7 How comfortable are you discussing the following gene therapies with a patient?

|  |  | | | |
| --- | --- | --- | --- | --- |
|  | Not comfortable: I could not answer questions or find more resources. (1) | Slightly comfortable: I could not answer questions in the moment but I would know where look up information after the session. (2) | Moderately comfortable: I could probably answer some questions or find more information for them. (3) | Very comfortable: I could probably answer many questions and point them to appropriate resources. (4) |
| Zolgensma to treat Spinal Muscular Atrophy (2) |  |  |  |  |
| Luxturna to treat RPE65 Associated Inherited Retinal Dystrophy (3) |  |  |  |  |
| Other (please describe) (not required) (4) |  |  |  |  |

| 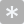 |
| --- |

Q8 How often do you directly discuss the following gene therapies with a patient?

|  |  | | | |
| --- | --- | --- | --- | --- |
|  | Never (1) | Rarely (once or twice a year) (2) | Sometimes (once or twice a month) (3) | Often (once or twice a week) (4) |
| Zolgensma to treat Spinal Muscular Atrophy (2) |  |  |  |  |
| Luxturna to treat RPE65 Associated Inherited Retinal Dystrophy (3) |  |  |  |  |
| Other (please describe) (not required) (4) |  |  |  |  |

Q9 Please summarize your familiarity with gene therapies and/or your experiences discussing them with patients (i.e. who initiates talking about gene therapy, in what context does it come up, what level of detail can you provide):

________________________________________________________________

________________________________________________________________

________________________________________________________________

________________________________________________________________

________________________________________________________________

End of Block: Experiences

Start of Block: Feelings, Attitudes, Experiences and Impact

Q10 How important do you feel it is for genetic counselors to know about available gene therapies for genetic conditions?

- Extremely important (1)
- Very important (2)
- Moderately important (3)
- Slightly important (4)
- Not important (5)

Q11 Which providers/individuals should feel comfortable discussing gene therapies with patients? Check all that apply:

- Genetic counselor (1)
- Geneticist (2)
- Pediatrician or general practitioner (3)
- Physician managing patients who potentially could be treated with gene therapy (ex: neurologist or ophthalmologist) (4)
- Patient support group/advocacy group (5)
- Other (please describe): (6) ________________________________________________

Q12 Do you feel that the availability of gene therapies has directly impacted your work/practice as a genetic counselor?

- Yes (1)
- No (2)

Skip To: Q14 If Q12 = No

Q13 Please describe how gene therapies have impacted your work/practice as a genetic counselor:

________________________________________________________________

________________________________________________________________

________________________________________________________________

________________________________________________________________

________________________________________________________________

Q14 As gene therapies become more readily available, what role do you see genetic counselors playing?

________________________________________________________________

________________________________________________________________

________________________________________________________________

________________________________________________________________

________________________________________________________________

End of Block: Feelings, Attitudes, Experiences and Impact

Start of Block: Education/Training

Q15 Did your genetic counseling program provide any training regarding gene therapies?

- Yes (1)
- No (2)
- Unsure (3)

Q16 Have you attended any trainings/continuing education hours regarding gene therapies?

- Yes (1)
- No (2)

Skip To: Q20 If Q16 = No

Q17 Who hosted the training(s)? Check all that apply:

- Drug manufacturers (1)
- A patient advocacy group (2)
- NSGC (3)
- Other professional organizational training (4)
- Training through your workplace (5)
- Word of mouth/discussions with colleagues (6)
- Popular media (TV, newspaper, magazines, etc.) (7)
- Scientific journal articles (8)
- FDA (9)
- Other (please describe): (10) ________________________________________________

Q18 What was the method of training(s)? Check all that apply:

- Brochure/written literature (1)
- Seminar/lecture (2)
- Sales pitch (3)
- Workplace discussion (4)
- Online training (6)
- Other (please describe): (5) ________________________________________________

Q19 Please briefly describe the training/educational experience:

________________________________________________________________

________________________________________________________________

________________________________________________________________

________________________________________________________________

________________________________________________________________

Q20 Would you be interested in additional training/education regarding gene therapies?

- Yes (1)
- No (2)

Skip To: End of Survey If Q20 = No

Q21 Who is the most appropriate provider for trainings/education? Check all that apply:

- Drug manufacturers (1)
- A patient advocacy group (2)
- NSGC (3)
- Other professional organizational training (4)
- Training through your workplace (5)
- Word of mouth/discussions with colleagues (6)
- Popular media (TV, newspaper, magazines, etc.) (7)
- Scientific journal articles (8)
- FDA (9)
- Other (please describe): (10) ________________________________________________

Q22 What method would you prefer for trainings/education? Check all that apply:

- Brochure/written literature (1)
- Seminar/lecture (2)
- Sales pitch (3)
- Workplace discussion (4)
- Online training (6)
- Other (please describe): (5) ________________________________________________

End of Block: Education/Training
